# Design of mechanically intelligent structures: Review of modelling stimuli-responsive materials for adaptive structures

**DOI:** 10.1016/j.heliyon.2024.e34026

**Published:** 2024-07-08

**Authors:** Qianyi Chen, Tarish Kalpoe, Jovana Jovanova

**Affiliations:** Department of Maritime and Transport Technology, Faculty of Mechanical Engineering, Delft University of Technology, Delft, 2628CD, the Netherlands

**Keywords:** Smart materials, Numerical approaches, Constitutive models, Model-based design, Non-linear behaviours

## Abstract

Smart materials are upcoming in many industries due to their unique properties and wide range of applicability. These materials have the potential to transform traditional engineering practices by enabling the development of more efficient, adaptive, and responsive systems. However, smart materials are characterized by nonlinear behaviour and complex constitutive models, posing challenges in modelling and simulation. Therefore, understanding their mechanical properties is crucial for model-based design. This review aims for advancements in numerically implementing various smart materials, especially focusing on their nonlinear deformation behaviours. Different mechanisms and functionalities, classification, constitutive models and applications of smart materials were analyzed. In addition, different numerical approaches for modelling across scales were investigated. This review also explored the strategies and implementations for mechanically intelligent structures using smart materials. In conclusion, the potential model-based design methodology for the multiple smart material-based structures is proposed, which provides guidance for the future development of mechanically intelligent structures in industrial applications.

## 1 Introduction

**Figure 1:**
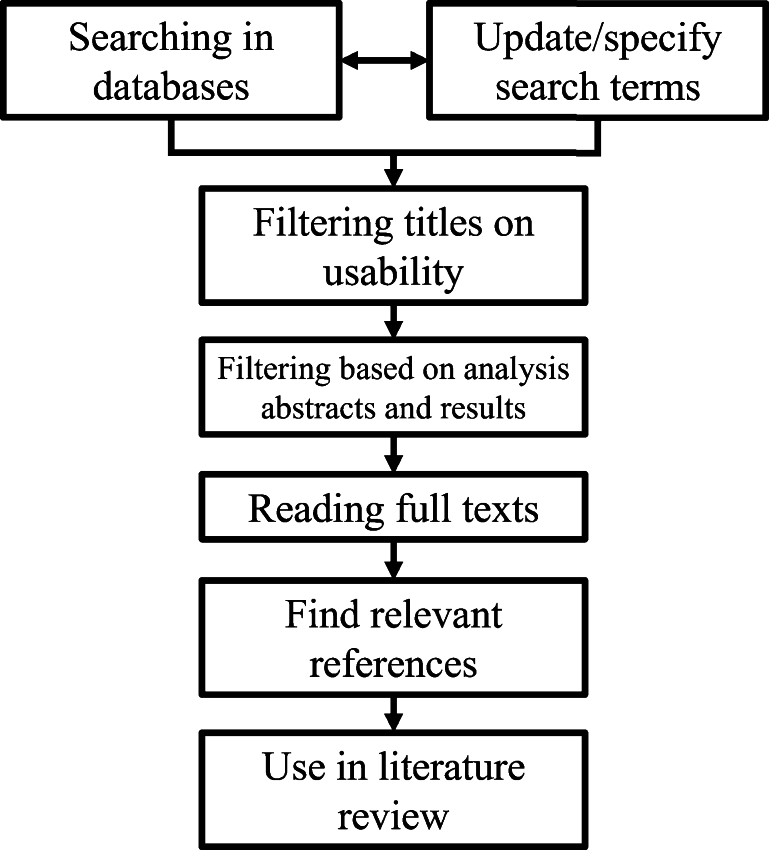
Schematic overview of the research strategy used.

One concept that has received significant attention in the vast and ever-changing landscape of engineering is the development and application of smart materials. These extraordinary materials can adapt and respond to changing environmental conditions, making them valuable in a wide range of applications [1]. Smart materials have emerged as a revolutionary force, offering unprecedented possibilities for innovation and advancement in many fields from aerospace and automotive engineering to electronics and biomedical devices [2], [3], [4], [5], [6], [7]. Smart materials have evolved into a needed aspect for a variety of structures due to their ability to adapt and modify their properties when subjected to external stimuli [8]. Unlike conventional materials, with fixed characteristics, smart materials can undergo reversible or irreversible changes in their physical or mechanical properties, enabling them to perform specific functions or tasks autonomously [9]. External stimuli including pH, stress, temperature, electric voltage and magnetic field can result in a change of size, viscosity or colour [10], [11]. Hence, the aforementioned parameters can be used to achieve tailored functions of the smart material's applications, such as sensors, actuators and medication delivery [12], [13], [14]. These materials have the potential to transform traditional engineering practices by enabling the development of more efficient, adaptive, and responsive systems [15]. Nevertheless, smart materials have several aspects which can be improved upon. The performance of these materials is susceptible to environmental conditions, some materials have a specific activation range and can not always function as efficiently when needed. Furthermore, durability issues with degradation and fatigue can reduce their lifespan. Additionally, these materials are often expensive due to their complexity in production and the need for specialized raw materials. Lastly, the implementation of these materials in existing systems can be challenging, requiring specialized expertise and has the potential for improvement with further research [1], [16], [17]. Therefore, the successful design and implementation of smart materials require a comprehensive understanding of their complex behaviour. Model-based design relying on solid modelling, finite-element analysis and optimization provides a potential solution, which makes the corresponding material and structure geometry selection easier [18]. Smart materials are meticulously modelled with advanced software tools suited for numerical simulations, facilitating the utilization of constitutive equation-based models [19]. Depending on the complexity of the model and various other considerations, such as the material's mechanical properties, these models are tailored to accurately describe nonlinear behaviours of smart materials [20], [21]. This study aims to provide an overview of different modelling techniques with fundamental constitutive equations. Literature is collected from different databases, such as but not limited to Scopus, SpringerLink and ScienceDirect. Literature has been studied from different aspects such as smart material types, modelling techniques and constitutive equations. The search strategy used in this review can be found in Fig. 1. An overview of the division of the found literature per category can be found in Table 1 with the respective reference numbers and an overview of the literature used in this review, sorted per year can be found in the diagram of Fig. 2. This article is structured as follows. It begins with an overview of the current classifications and types of smart materials. Then, the most common modelling techniques will be discussed. After this, the focus will be placed on the constitutive equations of shape memory alloys, shape memory polymers, hydrogel and magneto-rheological fluids. The review will be finished with a discussion and conclusion as well as the strategy for future research relating to the complicated modelling-based design of mechanically intelligent structures. The mechanically intelligent structure is the structure that is programmed to respond to the changes in the environments in a desired way with smart materials. Using model-based design to develop the design is a potentially efficient way to support the applications of smart materials in industrial engineering.

**Table 1:** Studied literature grouped per topic.

#	Topic	Literature
1	Smart materials in general	[1], [2], [3], [4], [5], [6], [7], [8], [9], [10], [11], [12], [13], [14], [15], [16], [17], [18], [19], [20], [21], [22], [23], [24], [25], [26], [27]
2	a) Chromic materials	[64], [65], [66], [67], [68], [69], [70], [71], [72], [73], [74], [75], [76], [77]
	b) Electric materials	[28], [29], [30], [31], [32], [33], [34], [35], [56], [57], [58], [59], [60], [61], [62], [63]
	c) Strictive materials	[36], [37], [38], [39], [40], [41], [42], [43], [44], [45], [46], [47], [48], [49], [50], [51], [52], [53], [54], [55]
	d) Shape memory materials	[78], [79], [80], [81], [82], [83], [84], [85], [86], [87], [88], [89], [90], [91], [92], [93], [94], [95], [96], [97], [98], [99], [100], [101], [102], [103], [104], [105], [106], [107], [108], [109], [110], [111], [112], [113], [114], [115], [116], [117], [118], [119]
	e) Rheological fluids	[120], [121], [122], [123], [124], [125], [126], [127], [128], [129]
3	Modelling approaches	[1], [19], [24], [74], [95], [130], [131], [132], [133], [134], [135], [136], [137], [138], [139], [140], [141], [142], [143], [144], [145], [146], [147], [148], [149], [150], [151], [152], [153], [154], [155], [156], [157], [158], [159], [160], [161], [162], [163]
4	Constitutive equations	[20], [80], [104], [105], [107], [140], [164], [165], [166], [167], [168], [169], [170], [171], [172], [173], [174], [175], [176], [177], [178], [179], [180], [181], [182], [183], [184], [185], [186], [187], [188], [189], [190], [191], [192], [193], [194], [195], [196], [197], [198], [199], [200], [201], [202], [203], [204], [205], [206], [207], [208]

**Figure 2:**
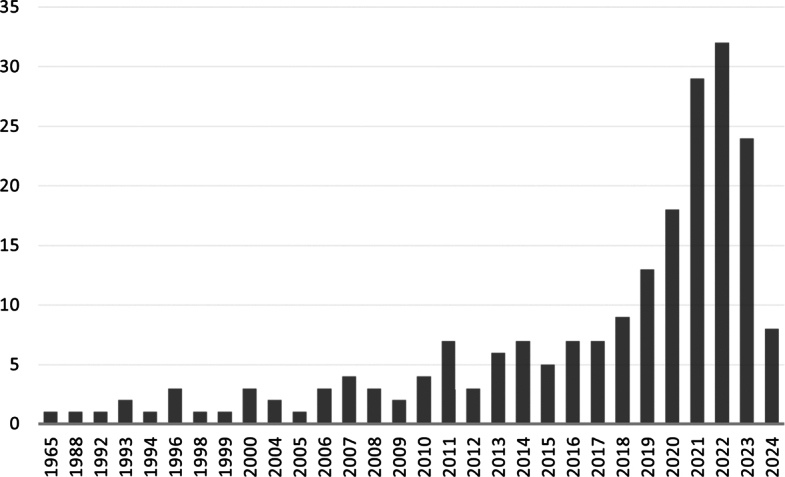
The amount of references used per year.

## 2 General aspects of smart materials

Materials fulfil different roles for several applications while taking costs and environmental influences into consideration. With aspects such as efficiency, innovation and safety being more prominent than ever before, smart materials are needed [22].

### 2.1 Classification of smart materials

Smart materials, also known as intelligent or responsive materials, are materials that exhibit adaptive or dynamic properties in response to external stimuli such as temperature, light, pressure, electric fields, or magnetic fields [23], [24]. These materials can be classified into several categories based on their underlying mechanisms and functionalities. The inner workings of smart materials are mimicking biological systems [25]. Smart materials gather information from their surroundings by sensing and creating chemical or physical effects for brain decision-making control. Smart materials are classified to provide a systematic framework for understanding and categorizing their unique properties and behaviours. Classification allows researchers and engineers to organize and differentiate smart materials based on their stimuli or response types, enabling a clearer understanding of their capabilities and potential applications. By categorizing smart materials, specific applications are achievable by tailoring the design and optimizing the performance of smart materials. Moreover, the classification of smart materials facilitates knowledge sharing and collaboration within the scientific community, for establishing a common language and reference point to understand smart materials.

The most significant distinction made in smart materials is between the main classes ‘active’ and ‘passive’ [1], [3]. Passive smart materials are responsive to external stimuli but do not actively generate a response. Instead, their properties change passively in reaction to specific environmental conditions. Passive materials can transfer energy, for instance when used as fibre optic [9], [26]. However, while being ‘smart’ they do not possess the characteristics to transduce energy. They are mostly used to function as a sensor instead of an actuator or transducer [27]. Subsequently, active materials can change their characteristics when exposed to external effects. Furthermore, some of the active smart materials can convert energy from one form such as thermal, mechanical or chemical to another form. Active smart materials possess some properties which distinguish them from other materials such as immediacy: short response time, self-actuation: form and shape can be changed due to external stimuli, self-diagnostic: imperfections or cracks can be detected and self-healing: materials can repair themselves when damaged.

### 2.2 Mechanism of smart materials

The main types of smart materials will be listed and elaborated on briefly in this section.

*Piezoelectric Materials*

Piezoelectric materials generate an electric charge when subjected to mechanical stress and, conversely, undergo deformation when an electric field is applied [28]. For a material to exhibit piezoelectric behaviour, the material needs to be dielectric, meaning nonconducting and without a centre of symmetry in the structure [29]. When an electric charge/voltage is applied as the response to mechanical forces/pressures, this is called the direct piezoelectric effect. In addition, when an electric charge is applied and leads to deformation, it is called the inverse piezoelectric field. Similarly, piezoelectric actuators can convert electrical signals into mechanical motions which is used commonly in the automotive industry. For instance in lenses and positioning of mirrors. They are also used in sensors and energy harvesting devices, such as flexible piezoelectric devices [30], [31], [32], [33]. Potential improvements for these materials can be found in their brittleness, many piezoelectric materials are ceramic, which are prone to cracking. Additionally, improving their performance under high temperatures could lead to more robust and reliable applications [34], [35].

*Photostrictive Materials*

Photostrictive materials undergo mechanical deformation when exposed to light. These materials use the combined effects of piezoelectric and photovoltaic properties present in the material. Through the photovoltaic effect, these materials produce an electric charge when exposed to light, and the piezoelectric effect causes strain or deformation [36], [37]. One of the most commonly studied photostrictive materials is BaTiO3 [38]. These materials have a distinct advantage of wireless actuation since no external electrical power sources are needed to cause mechanical changes. This makes these materials highly suitable for applications with remote control, such as microelectrochemical systems (MEMS), solar energy harvesting, actuators and optomechanical devices [39], [40], [41]. Photostrictive materials present opportunities for enhancement in efficiency and environmental resilience. Further research could focus on improving their performance under prolonged exposure to light and varying environmental conditions [42].

*Electrostrictive Materials*

Electrostrictive materials are a category of smart materials that experience a change in shape or size when subjected to an electric field [43]. This phenomenon, known as the electrostrictive effect, occurs due to the alignment of electric dipoles within the material under the influence of the electric field. As the dipoles align, the material undergoes mechanical strain, resulting in the dimensions changing [44]. One of the key characteristics of electrostrictive materials is the ability to respond rapidly to changes in the applied electric field. The precision and responsiveness of electrostrictive materials enable the development of advanced devices in robotics, aerospace, and biomedical engineering, including actuators, sensors, and tunable lenses [45], [46], [47]. Electrostrictive materials face difficulties in non-linearity, the response of these materials is often not linear. This makes control very hard in potential applications. Furthermore, hysteresis during operation can lead to energy losses, which leads to lower efficiencies [48], [49].

*Magnetostrictive Materials*

Magnetostrictive materials are a class of smart materials that exhibit mechanical deformation in response to changes in magnetic fields [50]. This phenomenon, known as the magnetostrictive effect, is characterized by the alignment and reorientation of magnetic domains within the material, leading to changes in its shape or size. Terfenol-D, comprised of terbium, dysprosium, and iron, stands as a prominently utilized magnetostrictive material [51]. Terfenol-D demonstrates significant magnetostrictive properties, making it a popular choice for various applications, including high-precision actuators, sensors, and vibration energy harvesters [52]. Magnetostrictive materials offer several advantages, such as high force output, fast response times, and excellent energy conversion capabilities. These properties make them ideal for applications where precise and efficient motion control is required, such as in robotics and adaptive structures [45], [53]. There are opportunities to improve these materials by addressing the formation of eddy currents at high frequencies, which can impede the excitation of the material core. Additionally, enhancing circuit design can help manage current leakage and demagnetization more effectively [54], [55].

*Thermoelectric Materials*

Thermoelectric materials are a class of smart materials that can convert heat into electricity or vice versa [56]. These materials exhibit the thermoelectric effect, where a temperature gradient across the material generates an electric potential, leading to the conversion of heat energy into electrical power [57]. The thermoelectric effect relies on the unique properties of thermoelectric materials, such as high electrical conductivity combined with low thermal conductivity. This combination allows them to efficiently transport electric charge while impeding the flow of heat, resulting in the creation of a temperature difference when one side of the material is exposed to heat [58]. Thermoelectric materials have gained significant attention in various applications due to their ability to harness waste heat and convert it into useful electrical power. They have potential uses in powering electronic devices, remote sensors, and even generating electricity from industrial processes or automotive exhausts [59], [60], [61]. Thermoelectric materials have the potential for an expanded operating range. Further research into the use of alloys could significantly enhance this aspect [62], [63].

*Electrochromic Materials*

Electrochromic materials are a category of smart materials that undergo reversible changes in their optical properties when subjected to an electrical stimulus [64], [65]. These materials are particularly desirable for applications such as smart windows, displays, and privacy glass because they can change colour or transparency in response to an applied voltage or current [66]. Electrochromic devices usually include several layers, including an electrochromic layer, an ion-conducting electrolyte, and electrodes. When an electric potential is supplied, ions migrate within the electrochromic layer, causing its optical characteristics to alter [67]. Electrochromic materials' capacity to alter light transmission and reflection makes them energy-efficient and adaptable, allowing them to provide benefits such as customizable shade, glare reduction, and privacy options in a variety of industries [68], [69]. These materials offer opportunities for enhancement by expanding the range of compatible materials. Additionally, increasing the spectrum of available colours would benefit applications requiring diverse colours, such as displays. Further research into hybrid materials could also improve their efficiency and compensate for their shortcomings [70], [71].

*Photochromic Materials*

Photochromic materials are a form of smart material that change their optical characteristics when exposed to light [72]. These materials can change colour or transparency when exposed to specific light wavelengths, making them valuable for applications like photochromic lenses, eyeglasses, and smart windows [73]. The presence of photochromic molecules inside the structure of photochromic materials is responsible for their behaviour. When these molecules absorb photons, they undergo a reversible chemical rearrangement, resulting in a change in their electronic configuration and a visible change in colour or transparency [74]. The primary advantage of photochromic materials is their ability to flip between different optical states in real-time. They enable automatic light adaptation by darkening when exposed to direct sunlight and reverting to their former condition when the intensity of the light drops [75]. Typically, chromic materials exhibit sensitivity to both visible and ultraviolet light, with colour variations contingent upon the intensity of the respective radiation spectrum. A prominent drawback of photochromic applications is poor durability and endurance. Free radicals produced by exposure to UV light play a big role in the degradation of these devices. Coatings could improve durability and provide protection to these devices [76], [77].

*Hydrogel*

Hydrogels are three-dimensional networks of polymer chains that can absorb and retain large amounts of water or other solvents. As smart materials, they exhibit significant swelling or shrinking in response to environmental factors such as temperature, pH, light, or the presence of certain chemicals [78]. Instead of making use of the Shape Memory Effect (SME), hydrogel makes use of the Shape Change Effect (SCE). This effect takes place when switching between two states is instant, or close to instant. Hydrogels may form via diverse physical interactions or chemical reactions, contingent upon the specific application requirements [79]. For example, a majority of hydrogel that exists of poly(nisopropylacrylamide) (PNIPAM) can swell and shrink in water due to temperature variations [80]. Even under pressure, the absorbed water can almost not be removed. Due to this property hydrogels are widely utilized in different fields. For instance, photoresponsive hydrogels are used to mimic the changing nature of biomechanics in living tissues [81]. Recently hydrogels have been used in electronics and electronic devices [82]. Hydrogels have applications in drug delivery, tissue engineering, and soft robotics [83], [84], [85], [86]. Furthermore, they are used as gel actuators, water-blocking tape in the field of biochemicals or agriculture engineering [87], [88], [89]. Key improvements can be made to make the material less highly sensitive to environmental conditions, reducing the altering of the properties of hydrogel. Further research could also be performed to reduce the deterioration, which is caused by repeated cycles of swelling and shrinking [90].

*Shape Memory Materials (SMMs)*

SMMs have the ability to “remember” their original shape and recover it when subjected to certain stimuli such as heat, stress, or magnetic fields, this mechanism is also known as the SME [91]. SMMs are one of the most commonly used intelligent materials for industrial purposes. SMMs have a lot of common groups such as shape memory Alloys (SMAs), shape memory polymers (SMPs) and shape memory ceramics (SMCs) [92], [93].

*(1) Shape Memory Alloys (SMAs)*

One of the common groups of SMMs is SMAs. These are metallic alloys with mechanical properties that alter with temperature. When heated, they revert to their previous shape by transitioning between stable crystalline phases such as martensitic or austenitic [94]. At high temperatures, the crystalline structure of the material is densely packed, having a hexagonal lattice (austenite). While at low temperatures, the structure is more loose having a body-centred lattice resulting in Martensite. SMAs have two distinct features, one being the SME and the other one being superelasticity (SE). Due to SE, the alloys can endure large strains, up to 8-10%, and still recover their original shape [95]. This effect happens at high temperatures and this results in the storage of energy. SME on the other hand provides motion and force. Looking at the reversibility of the SME, two common groups of SMAs can be found. The first one is a one-way SME and the other one is a two-way SME. The one-way SMAs have deformed to an irreversible state and the two-way SMAs are able to return to the original shape [96]. Nickel-titanium or copper-based alloys are commonly used to make SMAs. Few applications of SMAs can be found in industrial engineering [97]. SMAs are utilized to strengthen concrete beams in structural applications to decrease damage caused by corrosion, fire or mechanical loads [98]. In the aerospace sector, SMAs could be potentially used as sensors and controls [99]. In the marine industry, SMAs can be used in the shipyard for manufacturing and combining pipes [100]. Furthermore, applications can be found in the biomedical field as dental diagnosis or neurosurgical stents [101], [102]. Yet, SMAs also face difficulties in their potential applications. One of the biggest improvements can be made in the high costs associated with SMAs made from nickel-titanium, they are expensive and difficult to manufacture [1], [92].

*(2) Shape Memory Polymers (SMPs)*

A specific type of SMM is the SMP. SMPs maintain a permanent shape at room temperature, when exposed to a higher transition temperature they deform. After cooling down, the initial shape/form is obtained. Currently, the SME can be activated in SMPs by essentially three types of external stimuli, which consist of heat (thermo-responsive SMPs), chemicals (chemoresponsive SMPs) and the last one being light (photo-responsive and photo-thermal responsive SMPs) [103]. However, most SMPs are thermally responsive [104]. Currently, many studies have been conducted on one-way, two-way and multiple SMPs [105]. The first kind, one-way, can be traced in conventional crosslinked polymers [106]. Two-way SMPs are also known as reversible SMPs. This term refers to polymers which can deform at high temperatures and return between two shapes at low temperatures. The last of the three SMPs, multiple SMPs, are polymers which are able to memorize more than one temporary shape. The multiple-shape memory phenomenon enables the polymer to switch between different shapes by programming [107]. Applications are mostly affiliated with heat-shrinkable objects, such as heat-shrinkable tubes or labels. Examples of more high-tech devices include self-deployable hinges or biomedical devices, such as smart surgery devices [108], [109]. SMPs allow for more deformation compared to SMAs, lower costs, tunable stiffness and ease of fabrication. Recently, the mechanical properties have been improved enormously due to the reinforcement with other materials, leading SMPs to gain more attention [92]. Furthermore, new research on the effect of printing parameters on SMPs has been conducted, leading to a broader aspect for future applications [110].

Additionally, another aspect of printing SMPs worth mentioning is the rise of 4D printing, which is an advanced Additive Manufacturing (AM) process [111]. It is the process of a 3D-printed object is capable of changing or modifying its structure due to external influences, such as light or temperature. Therefore making smart or stimuli-responsive materials one of the most important components needed for 4D printing [112]. The big difference with standard 3D printing is that a new function is added to change shape over time. Making the structure capable of self-healing. For instance, in the case of a damaged pipe, the structure possesses the ability to self-heal. A specific example would be, the 4D printing of polyvinyl chloride (PVC) with good shape memory effects [113]. Another example would be self-assembling. This could take place when small components are moved through tiny holes in the human body, the structure will be able to assemble later for medical purposes when needed [114], [115], [116]. Nevertheless, SMPs are associated with lower structural properties, such as their mechanical strength. Therefore, incorporating a second material or creating a composite is often beneficial for most applications [92].

*(3) Shape Memory Ceramics (SMCs)*

The last common group of SMMs are SMCs. They provide many advantages compared to metallic alloys, such as higher strength and higher operating temperatures [117]. SMCs are less likely to degrade due to creep and oxidation at higher temperatures than SMAs. Compared to SMAs a higher actuation stress and strain can be exhibited, with a bigger transformation temperature range. Shape memory behaviour is seen by a reversible martensitic phase transformation. Out of the current SMCs, ceramics based on zirconia ($ZrO_{2}$) have gained a lot of interest, due to the similarity to SMAs regarding thermo-mechanical capabilities [118]. Ceramics that are zirconia-based consist of the largest family of SMCs with shape memory behaviour and mechanisms similar to SMAs [119]. Brittleness of SMCs can still be improved, since this would make the material more suitable for potential applications. Further research could also find a solution for the activation energy needed for SMCs, which is higher than for other SMMs [92].

*Electro-Rheological Fluids and Magneto-Rheological Fluids*

Electro-rheologic fluid (ERF) is a type of material that exhibits changes due to its ‘flow behaviour’ impacted by an applied electric field [120]. When an electric field is applied to a suspension of minuscule particles in an electrically insulating fluid, they rapidly form a structure which is similar to a solid, aligned in the direction of the electric field [121]. Depending on the application of the electric field, the initial state can be obtained such as gel or liquid, making it undergo rapid changes in viscosity [122]. ERFs can be very stiff, they have a high dielectric constant, and varying damping coefficient depending on the field and interfacial bond strength. ERFs are applied in vibration isolators, in automotive applications such as the clutch of shock breakers. In addition to this, they are also utilized in base-isolation for buildings and electro-active actuators, due to their ability to overcome imperfections caused by sedimentation and particle aggregation [123].

Magneto-rheologic fluid (MRF) is a type of smart material that exhibits changes in its rheology due to the application of a magnetic field. MRFs are two-phase composites consisting of solid particles which are magnetically polarizable and that are suspended in a non-magnetic medium. Once the magnetic field is turned on or applied, the materials transition from a Newtonian fluid to a solid state [124]. Due to this MRFs are also known as magneto-sensitive smart materials. Just like ERFs, MRFs were used commonly as dampers, buffers and clutches in the automotive industry. Specifically, MREs are still used regularly in vibration absorbers [125]. In recent years, MRFs are largely used for finishing purposes in the manufacturing industry. MRFs combined with abrasive particles are used as a finish, since using fluid is an efficient method to achieve manipulation [126]. MRFs are similar to ERFs with varying viscosity depending on the field applied. The viscosity when a magnetic field is applied, is several orders higher than in its fluid state [127], [128]. One area for enhancement in these rheological fluids is their tendency to thicken over time, necessitating periodic replacement. Additionally, their high density presents an opportunity to innovate towards more lightweight applications [129].

**Figure 3:**
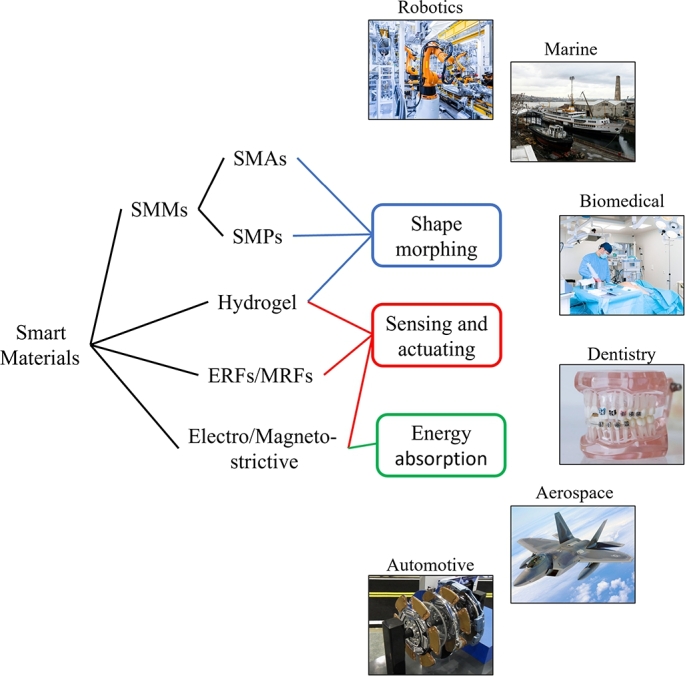
Potential applications of the main types of smart materials.

The field of smart materials is diverse and continuously evolving, with new materials and functionalities being discovered and developed. Overall, the classification of smart materials promotes efficient utilization, integration, and advancement in various industries. The mentioned examples can be found in Table 2 among others with their respective outputs and inputs. In Fig. 3 some applications can be found regarding the main classes mentioned.

**Table 2:** Smart material types with respective inputs and outputs.

Material		Input	Output
Piezoelectric		Electric field Mechanical load	Mechanical strain Electric potential
Photostrictive		Incident light	Mechanical strain
Magnetostrictive		Magnetic field Mechanical load	Mechanical strain Magnetization
Electrostrictive		Electric field Mechanical load	Mechanical strain Electric potential
Thermoelectric		Thermal load Electric field	Electric potential Temperature change
Shape memory materials		Thermal load Magnetic load	Mechanical strain
Photovoltaic		Incident light	Electric potential
Magneto- Electro-	} rheological fluids	Magnetic field Electric field	Mechanical strain
Photo- Thermo- Magneto- Electro-	} chromic	Incident light Thermal load Magnetic field Electric field Mechanical load	Color change

## 3 Modelling techniques

In recent years, major developments have been made in computer science and in particular simulations. Computer simulations have become more relevant in the development of new materials. As the demand for modelling and simulation grows, they are not only used for system analysis but also for designing and optimization. The relevance of modelling is discussed here below referencing different aspects concerning smart materials [130]:

Understanding Material Behaviour: Smart materials exhibit unique properties and behaviours that can be difficult to predict and control without proper modelling. By developing accurate models, engineers can gain insights into the underlying mechanisms and behaviour of smart materials, enabling them to design more effective systems [95].

Performance Optimization: Modelling allows engineers to explore and optimize the performance of smart material systems before physical prototyping. By simulating the behaviour of the materials and their interaction with the surrounding environment, designers can identify the optimal configuration and dimensions to achieve desired performance objectives [131].

Cost and Time Savings: In a lot of cases, it is not feasible to assemble an experimental setup or configuration due to costs or environmental constraints. Building physical prototypes of smart material systems can be time-consuming and expensive. By using models, engineers can quickly evaluate different design alternatives and iterate on them virtually, reducing the need for multiple physical prototypes and associated costs [132].

Risk Mitigation: Smart materials often have complex responses and can exhibit nonlinear behaviour. By using models, engineers can analyze and predict how the material will behave under various operating conditions and external stimuli. This enables them to identify potential risks and mitigate them in the design stage, improving overall system reliability [1].

Scalability and Generalization: Since smart materials are often utilized in various applications, models provide a framework for capturing the fundamental behaviour of smart materials, allowing their application in different systems and scenarios. Once a reliable model is developed, it can be used as a basis for designing a wide range of smart material-based systems, saving time and effort in each new application [24].

Overall, modelling empowers engineers to understand, optimize, and control the behaviour of smart materials, leading to more efficient and reliable systems while reducing development time and costs. The specific modelling approach can vary widely depending on the type of smart material and the intended application. Advanced numerical tools and specialized modelling techniques are often employed to accurately capture the behaviour of these materials [133], [134], [135].

### 3.1 Finite element analysis (FEA)

FEA is based on the Finite Element Method (FEM), which is unquestionably one of the most commonly used numerical tools for understanding physical systems with complicated boundary conditions [136], [137]. Therefore, FEA is an efficient approach to investigate the nonlinear behaviours of smart materials. Due to its broad application, the implementation of constitutive equations in FE software, such as COMSOL, ABAQUS and ANSYS has been gaining a lot of attention [114], [138]. Modelling smart materials using FEA involves several steps [139], [140]:

For each material or structure the geometry needs to be defined. The geometry is discretized into smaller elements by meshing. The material properties need to be specified and will depend on the type of smart material being modelled. For instance, for SMMs, phase transformations would be needed in combination with corresponding temperatures and mechanical properties.

To model industrial scenarios appropriate boundary conditions need to be applied. This consists of fixing nodes/surfaces, applying loads and adding thermal/electrical boundary conditions. A suitable solver needs to be chosen within the FEA algorithm since they can vary in terms of speed and accuracy. Smart materials often have complex constitutive models that relate various physical parameters. It is important that FEA algorithm supports the constitutive equations for integration and implementation [19].

The software/solver will solve the equations governing the material behaviour of the smart material based on the given material properties, boundary conditions and geometry. In addition, convergence of the solution is essential to establish stability and acceptability. Furthermore, data visualization through plots and quantitative data extraction are imperative for analysis. Subsequently, comparison with experimental data will validate the results, prompting adjustments in parameters. Finally, conducting sensitivity analysis will explain the impact of material properties and other parameters on the desired behaviour within the model [141].

The accuracy of the FEA model depends on the fidelity of the material properties, the mesh quality, the chosen solver, and the adequacy of boundary conditions. The physics associated with the model needs to be included as accurately as possible to create a precise model. For instance, FEM is also used to account for nonuniformities of the electric field used to model actuation performance. This is an approach useful for dynamic modelling with enhanced robustness [142], [143]. Additionally to the FEM, other numerical methods are also known as alternatives for FEA.

Boundary Element Method (BEM): This method is based on an integral equation formulation of a boundary value problem. BEM is more useful for concise 3D objects but has more difficulties with vast geometries. This method is more focused on the boundary instead of the entire geometry [144], [145].

Finite Difference and Finite Volume Methods: These methods also make use of a boundary value problem, but instead of the integral equation formulation, the focus lies on the differential formulation. They are mostly used in computational fluid dynamics and heat transfer scenarios, serving as effective tools for conserving laws of physics [146], [147].

### 3.2 Multiscale modelling

**Figure 4:**
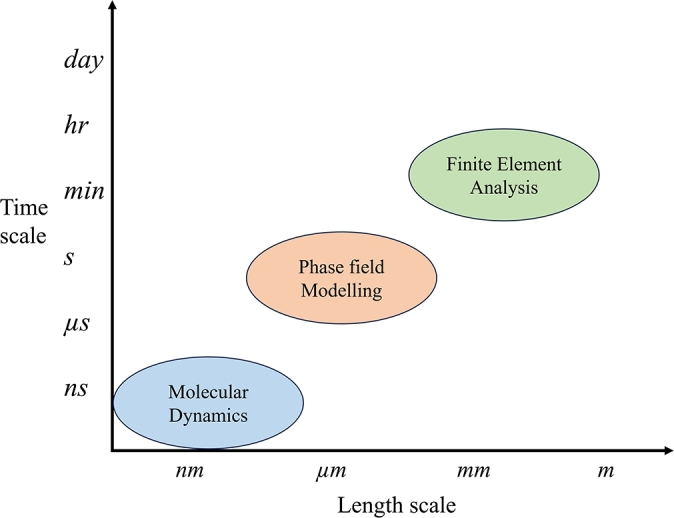
Different types of length and time scales associated with the methods of simulations.

Smart materials' intricate microstructures affect their behaviour, making multiscale modelling a vital method for investigating both macroscopic and microscopic phenomena in these materials [148]. Stimuli-responsive polymers are the types of smart material where Multiscale modelling would seem more suitable. By looking at different scales a broad scope of the material could be researched. Each length scale is also associated with a different simulation method, which can be seen in Fig. 4 [149], [150]. In this section three simulation methods of Multiscale modelling are briefly discussed based on different scales applicable to stimuli-responsive polymers [134], [151].

*All-Atom Molecular Dynamics (AAMD)*

AAMD is a method used for studying the characteristics of polymers at an atomic level. This method calculates particle locations over time using the classical Newton's equation of motion. The time scales of AAMD are typically in the tens to hundreds of nanoseconds range, with length scales in the tens of nanometers [152]. AAMD is mostly used to explore the response of the stimuli-responsive polymers. This technique regulates the interactions between particles by using a special energy function, often known as a force field, which consists of bonded (bondangles, bondlengths, etc.) and non-bonded components (van der Waals) parameters [153].

*Coarse-Grained Molecular Dynamics (CGMD)*

For comparatively bigger polymer systems, AAMD will be constrained by computing time and resources. It is not suitable for use in bigger polymer systems. To address this issue, much work has been put into developing particle-based coarse-grained models to investigate large systems [134]. CGMD simulations can help bridge the gap between atomistic simulations and experiments in the understanding of polymers across the range from micron scale [154], [155]. In CGMD, relating to smart polymer modelling, the system can be simplified as each molecule of the polymer is coarse-grained into a string of beads, which are all connected. It is represented as a sphere of determined mass repelling each other via the Weeks-Chandler-Anderson potential [156]. In addition to this, simulation results found with AAMD and experimental results are used to gain relevant force field parameters.

*Dissipative Particle Dynamics (DPD)*

The DPD approach, aligns with the time scale of the response of self-assembled polymers. This is a mesoscopic simulation method, with mesoscopic fitting between macroscopic and atomic [157]. This method has been utilized as a prominent tool to research complex systems of soft matter, therefore it can be used to study the stimuli-responsiveness of polymers. In DPD simulations, polymer monomers will be simplified to DPD beads by coarse-graining. Compared to the AAMD method, the atomic clusters are replaced by dissipative beads, with the details just being included as internal forces [158]. In DPD simulations, physical quantities are often expressed in reduced units for computational simplicity. Similar to the AAMD approach, bead motion is determined by solving Newton's equations of motion [159].

*Phase Field Modelling (PFM)*

The arrangement and characteristics of different phases on a microscopic level have a major influence on the behaviour of materials on the macroscopic level [160]. The imperfections, or defects, in the lattice, determine the properties of most materials. Defects such as dislocations, precipitates or solute atoms in the lattice, but also defects in the lattice boundaries, such as grains. The microstructure-properties relationship is commonly explored using FEM. However, FEM struggles with dynamic boundary changes during processing. PFM provides a robust alternative for simulating microstructural evolution [161], [162].

PFM is particularly relevant for phase transformations and complex changes in microstructure over time under the influence of factors, such as stress, temperature and composition. For instance, this is important for SMAs since a transition is made from austenite to martensite. The key principle behind PFM is making use of continuous fields for the interfaces. All the discontinuities found across the interfaces, in addition to some boundary conditions, are depicted by a clean smooth version of a phase field. Every PFM is based on this formulation of the free energy as a functional [163]:

(1) $$F=\int f(\phi_{1},\phi_{2},...,\phi_{n},c_{1},c_{2},...,C_{n},\nabla \phi_{1},\nabla \phi_{2},...,\nabla \phi_{n},\nabla c_{1},\nabla c_{2},...,\nabla C_{n},p,T,...)$$

with *ϕ* being a set of non-conserved fields and *c* a set of conserved fields. The energy function *f* normally includes a potential with local minima, gradient terms such as ∇*c* which relate to the energetic costs and state variables such as pressure and temperature, or other external stimuli which is the case for most smart materials.

## 4 Constitutive models for smart materials

Implementing constitutive models is a crucial step in using numerical methods [20], [140]. Solving complex problems in materials science and engineering requires modern numerical methods, but their effectiveness hinges on the quality of the constitutive models [164], [165]. These models describe the mathematical relationships between the material's behaviour of different factors such as stress, strain, etc. The mathematical relationships are found to be the constitutive equations, which are essential for understanding and predicting how materials will behave under various loading conditions [166]. Constitutive equations can range from linear relationships to more complex nonlinear equations, such as to account for large deformations at high temperatures. Constitutive equations are frequently derived from experimental data and can be application-specific. Therefore, the exact form of these equations depends on the material and the phenomena that are dealt with [167], [168]. This section is dedicated to conclude the common constitutive equations for SMAs, SMPs, hydrogels and MRFs. In addition, the review presents the constitutive models of various smart materials, emphasizing their properties and state changes, without delving into dynamic analysis.

### 4.1 Constitutive models for SMAs

Due to external stimuli caused by the change of the microscopic phase transformations of the phases austenite and martensite, SMAs have the macroscopic mechanical properties of the SME and SE. The stress-strain constitutive model encompasses both complex mechanical properties and phase transformations [169].

*Adjusted Brinson Model*

The behaviour of the SMA material relies on a set of variables such as the stress, temperature and the crystal structure, the latter depends on the loading and thermal histories. The constitutive model is based on loading (tensile force) at a low temperature, unloading and then heating [170]. To account for this, in SMAs an internal variable is introduced *ξ*. This letter depicts the condition of the materials as a martensite material fraction, with ξ=0 indicating the material is completely in the austenite phase. The created martensite has two results, twinned or detwinned [171]. Twinned indicates multiple martensite variants coexisting, while detwinned suggests a connection with one dominant variant, as expressed in Equation (2),

(2) $$\xi =\xi_{S}+\xi_{T}$$

where the subscript *S* stands for stress-induced and *T* for temperature induced. The model is an adjusted model for the situation of a simple one-dimension SMA model proposed by Brinson [172] and is given in Equation (3),

(3) $$\sigma -\sigma_{0}=E(\xi )\varepsilon -E(\xi_{0})\varepsilon_{0}+\Omega (\xi )\varepsilon_{S}-\Omega (\xi_{0})\varepsilon_{S0}+\Theta (T-T_{0})$$

with *σ* depicting the stress currently resisted in the material, E(ξ) is the elastic modulus depending on the current crystal structure, *ε* is the strain experienced by the material and Θ is the thermal response shown by the material. All the variables with the “0” subscript indicate the initial state.

#### 4.1.1 Macroscopic models

The macroscopic models depict the behaviour of SMAs based on phenomenological considerations, simple micromacro thermodynamics or experimental data. The mentioned models in this section are pioneering models which are used as foundations for specific applications of models made for SMAs. These models have attributed to major developments in the modelling of SMAs [173].

*Boyd and Lagoudas - Phenomenological Model*

This model is based on an energy potential which is taken from physical considerations [174]. In this case, the state equation of this model is derived from the following Gibbs free energy potential [175], with $\psi_{A}$ and $\psi_{W}$ being the free energies of austenite and martensite. $\psi_{mix}$ indicates the mixing term reflecting on the different interactions between the two phases. This is the first model that takes into consideration the reorientation of martensite by including an inelastic strain tensor,

(4) $$\Psi \left(\sigma ,T,\xi ,\varepsilon^{\mathrm{tr}}\right)=(1-\xi )\Psi_{A}(\sigma ,T)+\xi \Psi_{M}(\sigma ,T)+\Psi^{\mathrm{mix}}\left(\xi ,\varepsilon^{\mathrm{tr}}\right),$$

the following stress-strain relation of Equation (5) is used in this model,

(5) $$\sigma ={\left[\xi S_{M}+(1-\xi )S_{A}\right]}^{-1}:\left[\varepsilon -\varepsilon^{\mathrm{tr}}-\alpha \left(T-T_{0}\right)\right]$$

*Auricchio and Petrini - Phenomenological Model*

This model is based on the Helmholtz free energy function and is written as a function of the total strain *ε*, $e^{el}$ and $e^{tr}$ being the elastic and transformation strain of the SMA. In addition to this *G* is the shear modulus and *κ* is the bulk modulus [176]. The model can be found below as Equation (6),

(6) $$\Phi \left(\varepsilon ,\varepsilon^{\mathrm{tr}},T\right)=\frac{1}{2}\kappa {\left(\mathrm{Tr}(\varepsilon )\right)}^{2}+G{\left\|e^{\mathrm{el}}\right\|}^{2}+\tau_{m}(T)\left\|e^{{\mathrm{tr}}^{r}}\right\|+\frac{h}{2}{\left\|e^{\mathrm{tr}}\right\|}^{2}+I_{0,\varepsilon_{\max}^{\mathrm{tr}}}\left(\varepsilon^{\mathrm{tr}}\right)$$

The associated stress-strain function found can be found below as Equation (7),

(7) $$\sigma =2G\mathrm{dev}\;\left(\varepsilon -\varepsilon^{\mathrm{tr}}\right)+\kappa \left[\mathrm{Tr}(\varepsilon )-3\alpha \left(T-T_{0}\right)\right]$$

#### 4.1.2 ZM phenomenological model

This model is based on an energy potential which is taken from physical considerations. In this case, the state Equations of this model are derived by Zaki and Moumni [177] from the Helmholtz free energy potential which is shown in Equation (8),

(8) $$\Phi \left(T,\varepsilon_{A},\varepsilon_{M},\varepsilon^{\text{ori }},\xi \right)=(1-\xi )\Phi_{A}+\xi \Phi_{M}+I_{\mathrm{AM}}$$

Leading to Equation (9) which is a strain-stress relationship, with $K_{a}$ as the elastic stiffness tensor of austenite and $K_{m}$ is the elastic stiffness tensor of martensite. This model is based on the assumption that the reversible inelastic deformation of SMAs is due to the orientation of the different Martensite forms,

(9) $$\sigma ={\left[(1-\xi )K_{A}^{-1}+\xi K_{M}^{-1}\right]}^{-1}:\left(\varepsilon -\xi {\bar{\varepsilon }}^{\mathrm{tr}}\right)$$

The above-mentioned models have in recent years been expanded, and more details have been added, which is based on the applications needed [178], [179]. Therefore, the initial models are added here since most of them have been adaptations of the original models.

### 4.2 Constitutive models for SMPs

The constitutive models are based on the investigation of the mechanical behaviour and the mechanisms associated with SMP structures, to depict the SME in as much detail as possible. Compared to other SMMs, offer the widest range of stimuli for activation [105], [107]. Even though different types of stimuli triggered SMPs work differently, the internal mechanism is similar to thermal-induced SMPs. Consequently, most research is dedicated to thermal-induced SMPs [180]. Many approaches exist to examine the principles and mechanisms of the shape memory phenomenon, such as the stress-strain relationship under several factors such as temperature. In general, there are two categories: rheological models, emphasizing viscoelasticity theory, and micromechanical models, centring more on phase transitions. Subsequent discussion will scrutinize these models [181], [182].

#### 4.2.1 Rheological models SMPs

Rheological models have been modelled as springs and dashpot elements. Due to the model parameters varying in temperature, the models can quantify the SME of SMPs. These models can efficiently analyze mechanical qualities affected by temperature, time, strain rate, and other factors.

*Basic Viscoelastic Model*

A simplified three-element model is proposed by Li et al. [183] to effectively simulate the characteristics of stress, strain and temperature in combination with a Kelvin element and a spring element all in series. These elements represent viscoelasticity, supplemented by accounting for heat expansion caused by temperature fluctuations. Consequently, the model delineates thermo-viscoelastic behaviour, derived from one-dissension constitutive equations, resulting in Equation (10),

(10) $$\sigma +\frac{\mu (T)}{\left(E_{1}(T)+E_{2}(T)\right)}\frac{d\sigma }{dt}=\frac{\mu (T)}{\left(1+E_{1}(T)/E_{2}(T)\right)}\left(\frac{d\varepsilon }{dt}-\alpha \frac{dT}{dt}\right)+\frac{1}{\left(1/E_{1}(T)+1/E_{2}(T)\right)}\left(\varepsilon -\alpha \left(T-T_{0}\right)\right)$$

where *σ*, *ε* and *T* indicate the stress, strain and temperature. The *α* depicts the expansion coefficient and *E* is the elastic modulus. The model presented in Equation (10) can efficiently predict the response of the SMPs by making use of simplified parameters, which is very beneficial for some applications in engineering.

*Thermoviscoelastic Models of SMPs coupled with temperature and rate effects*

The previous model has provided a simple method to depict the mechanical properties of SMPs, but only focuses on infinitesimal strains. Diani [184] has developed a model to account for large strain deformation. This model is based on thermodynamics, thus the deformation is based on internal energy and entropy. The total Cauchy stress has been formulated as follows in Equation (11),

(11) $$\begin{array}{c} \sigma^{\eta }=\frac{E^{r}}{6}\frac{T}{T_{h}}F^{e}F^{eT}-pI\\ \sigma^{U}=L^{e}\left[\ln\left(V^{e}\right)\right] \end{array}$$

with $\sigma^{\eta }$ indicates the stress from the entropy branch, with $T_{h}$ being a higher temperature than regular *T*. ${\text{F}}^{e}$ is the deformation gradient, *p* is the Lagrange multiplier and **I** is the invariant of the right Cauchy-Green tensor of the elastic deformation. With $\sigma^{U}$ indicating the stress from the internal energy branch, where ${\text{L}}^{e}$ is the elastic constant tensor and ${\text{V}}^{e}$ is the left stretch tensor found from the deformation gradient ${\text{F}}^{e}$. In general, this model can precisely make an estimation of the remaining strain when the stress is released. It provides a useful groundwork for the thermomechanical response of SMPs while experiencing large deformation.

*Fractional Viscoelastic Constitutive Model*

A majority of SMPs are described by relaxation behaviour, leading to a large number of material parameters to be determined experimentally. An attempt is made to describe the complex viscoelastic behaviour with a lower number of parameters, which can be seen in Equation (12). The fractional viscoelastic model is applied to accurately look into the thermomechanical responses of SMPs. The thermal expansion is considered independent of the mechanical behaviours and was defined separately from the model. For the complete derivation and parameters included refer to the work of [185],

(12) $$\begin{array}{c} \sigma_{M}(t)=\sigma_{\mathrm{eq}}(t)+\sum_{i=1}^{m}\sigma_{i}(t)=E_{\mathrm{eq}}\varepsilon_{M}(t)+\\ \sum_{i=1}^{2}[E_{i}\int_{t_{0}}^{t}E_{\beta }\left(-{\left(\frac{\left(t-\xi \right)}{\tau_{i}}\right)}^{\beta }\right)\frac{d\varepsilon_{M}(\xi )}{d\xi }d\xi +E_{\beta }\left(-{\left(\frac{t-t_{0}}{\tau_{i}}\right)}^{\beta }\right)\sigma_{i}\left(t_{0}\right)] \end{array}$$

where $\sigma_{M}$ is the total mechanical stress, *E* is the stiffness of the spring, $E_{\beta }$ is the Mittag-Leffler function [186]. *n* belongs to the set of integers, *τ* is the relaxation time and *t* is the time.

#### 4.2.2 Phenomenological models SMPs

Most phenomenological models for SMPs are built on the “meso-mechanical approach”, which simplifies the structure to continuous phases. Certain external stimuli lead to the different phases transforming into one other, which again reflects the properties of SMP. In comparison to the rheological models, the phenomenological models can relate the phase transitions to the SME, which provides a clear comprehension of the deformation mechanisms.

*Classic Phenomenological Constitutive Model*

The first phenomenological 3D model for thermally activated SMPs is proposed by [187], which suggested that the SMP structure consists of two phases, the frozen phase and the active phase. The deformation in the frozen phase is dominated by internal energy change, and polymer conformation motion occurs in the active phase. The frozen phase is the most important phase of a polymer in its glassy state, whereas the active phase is primarily concerned with the rubbery state. The classic phenomenological constitutive model decomposes the strain energy into thermal, elastic strain, and frozen entropic (stored) strain components and uses the frozen volume fraction as an internal variable to describe the microstructure evolution. All the parameters lead to the constitutive equation for SMPs in a thermomechanical cycle in one-dimension form which is expressed as follows in Equation (13),

(13) $$\sigma =\frac{\varepsilon -\varepsilon_{s}-\varepsilon_{T}}{\frac{\phi_{f}}{E_{i}}+\frac{1-\phi_{f}}{E_{e}}}=E\left(\varepsilon -\varepsilon_{s}-\int_{T_{h}}^{T}\alpha dT\right)$$

with $\phi_{f}$ being the frozen fraction, $\varepsilon_{s}$ frozen entropic strain, $\varepsilon_{T}$ the thermal strain, $E_{i}$ is the modulus associated with the glossy state at a low temperature and $E_{e}$ is the modulus associated with the entropic deformation at the rubbery state. For the complete derivation and parameters included refer to the work of [187].

*Phenomenological Models of SMP with Rate Effect*

Following the classic phenomenological constitutive model, a new model was proposed by [188]. The new model was a phase-evolution-based thermomechanical model aiming to describe the behaviours of amorphous SMPs. Equations were derived to describe the mechanical behaviours taking place within a complete thermal mechanical cycle [188]. By making use of the concept of frozen strain, which is dependent on time and temperature, Equation (14) can be derived:

(14) $$\begin{array}{c} \sigma_{\text{total }}=\frac{\varepsilon_{\text{total }}-\varepsilon_{f-\text{ real }}-\varepsilon_{T}}{{\frac{\gamma }{E}}_{g}}+\frac{1-\gamma }{E_{r}}\\ \frac{d\varepsilon_{f}}{dT}=\frac{d\gamma }{dT}[1-f(T)]\frac{\varepsilon_{\text{total }}-\varepsilon_{f}-\varepsilon_{T}}{E_{r}\left(\frac{\gamma }{E_{g}}+\frac{1-\gamma }{E_{r}}\right)}\\ \varepsilon_{f-\text{ real }}=\varepsilon_{f}-\int_{0}^{t}{\overset{˙}{\varepsilon }}_{f}e^{\frac{-(t-a)}{\tau }}da \end{array}$$

with $\sigma_{total}$ being the total stress, $\varepsilon_{total}$ is the total strain, $\varepsilon_{f-real}$ is the real frozen strain depending on the time, *γ* is the volume fraction of the glassy phase, $E_{g}$ is the elastic modulus in the glassy phase, $E_{r}$ is the elastic modulus in the rubbery phase, $\varepsilon_{f}$ is the total frozen strain in the materials. The new proposed model is fit to be used for predicting strain and stress respondents of SMPs under a free strain condition but is also capable of reproducing the behaviour of SMPs under other external constraints.

### 4.3 Constitutive models for hydrogel

A generalized theory has been created to specify different kinds of hydrogel, even if the hydrogels differ by their stimulus. This section gives the constitutive equations for different kinds of hydrogel, such as neutral gel, pH-sensitive gel, temperature-sensitive gel and photothermal-sensitive gel. The general model is the same, but the model has been adjusted for each of the gels concerning their properties. The constitutive equations make use of the Neo-Hookean hyperelastic material model [189], [190], whose strain-energy function is given in Equation (15) where *T* is the absolute temperature, $k_{B}$ is the Boltzmann constant, *N* is the crosslink density, *λ* is the principles stretch in each direction. This equation is chosen since hydrogels are a hyperelastic material type.

(15) $$W=\frac{1}{2}NK_{B}T(\lambda_{1}+\lambda_{2}+\lambda_{3}-3)$$

*Neutral Gel*

The most frequently studied type of gel, is the neutral gel. The neutral gel has one single stimulus and that is external exposure to water. Since there are no other stimuli, the constitutive model is fundamental. By combining the Flory–Rehner free-energy function due to stretching a network of polymers with the three principal normal stresses [191], the general form can then be expressed as follows in Equation (16),

(16) $$\frac{s_{iK}}{k_{B}T/v}=Nv\left(F_{iK}-H_{iK}\right)+\left[J\log\left(1-\frac{1}{J}\right)+1+\frac{\chi }{J}-\frac{\mu }{kT}J\right]H_{iK}$$

Equation (16) connects the stress component to the deformation gradient in the case of the gel being held at a not-varying chemical potential by a reservoir of solvent molecules. With $s_{iK}$ being one of the three nominal stresses, *v* is the specific volume, $F_{iK}$ is the deformation gradient, *μ* is the chemical potential, *J* is the volumetric strain, and *χ* is the dimensionless measure of the enthalpy of mixing [192].

*Salt Concentration Sensitive Gel*

When exposed to an ionic solution, polyelectrolyte networks absorb it, causing gel swelling, resulting in a salt concentration-sensitive gel [193]. Similar to the neutral gel, salt concentration sensitive gel is subjected to the same concentrations only with the stimulation of a salt concentration. Equation (17) describes the mechanical deformation of the salt concentration gel, including the free swelling and contained swelling cases. It combines the free energy functions of salt and ions with that of the stretching of the hydrogel. With $\sigma_{ij}$ being the true stress, J is the det(**F**), $C^{0}$ is the fixed charge in the gel, $c_{0}$ is the concentration salt, $C^{+}$ and $C^{-}$ are the positive and negative ion concentrations [194].

(17) $$\begin{array}{c} \frac{\sigma_{ij}}{\frac{kT}{v}}=\frac{Nv}{J}\left(F_{iK}F_{jK}-\delta_{ij}\right)+\left(\log\frac{J-1}{J}+\frac{1}{J}+\frac{\chi }{J^{2}}+2\nu c_{0}\right)\delta_{ij}+\\ \nu \delta_{ij}\cdot \\ {}[c^{+}\left(\log\frac{c^{+}}{c_{0}}-1\right)+c^{-}\left(\log\frac{c^{-}}{c_{0}}-1\right)+J\left(\frac{\partial c^{+}}{\partial J}\log\frac{c^{+}}{c_{0}}+\frac{\partial c^{-}}{\partial J}\log\frac{c^{-}}{c_{0}}\right)] \end{array}$$

It is worth noting that during the last decade, studies have disagreed if the concentration $C^{a}$ is dependent on *J*. In the derivation resulting in Equation (17), it is assumed that it is dependent to realize an equation which can be implemented in FEM. For the detailed derivation with all the assumptions please refer to the study of [194].

*pH-Sensitive Gel*

The pH-sensitive hydrogel consists of a network of stretching soft materials which have acidic groups and are in equilibrium with a solution of which the solvent is water and mechanical forces [195]. The constitutive equation is based on the summation of the free energies of the stretching polymers, the solvent, the ions and the acidic groups: *W* = $W_{str}$ + $W_{mix}$ + $W_{ion}$ + $W_{dis}$. Equation (18) emerges from this summation, where $c^{H^{+}}$, ${\overset{¯}{c}}^{-}$, and ${\overset{¯}{c}}^{+}$ denote the concentrations of protons, negative ions, and positive ions in the external solution, respectively. For a detailed derivation, refer to the work of [196].

(18) $$\frac{\sigma_{ij}}{kT/v}=\frac{Nv}{J}\left(F_{iK}F_{jK}-\delta_{ij}\right)+\left(\log\frac{J-1}{J}+\frac{1}{J}+\frac{\chi }{J^{2}}\right)\delta_{ij}\;\;-\nu \delta_{ij}[c^{H^{+}}+c^{+}+c^{-}-{\overset{¯}{c}}^{H^{+}}-{\overset{¯}{c}}^{+}-{\overset{¯}{c}}^{-}]$$

*Temperature-sensitive Gel*

Temperature-sensitive hydrogels, also known chemically as PNIPAM hydrogels, are hydrogels that can withstand large changes in volume owing to temperature changes [80], [197]. Due to their distinctive characteristic of exhibiting a sharp macromolecular transition from hydrophilic to hydrophobic, temperature-sensitive hydrogels constitute a significant branch within the realm of hydrogels. In addition to this, these types of hydrogels are transparent, elastic and flexible. The constitutive equation is similar to the one of neutral gel, the only addition is the interaction parameter *χ* with $\chi_{0}$, $\chi_{1}$ and *ϕ* (Equation (19)) being dependent on experimental data obtained for PNIPAM [198].

(19) $$\chi =\chi_{0}+\chi_{1}\cdot \phi$$

By combining Equation (16) of the neutral gel with Equation (19) and the uniaxial load case the following two lines can be obtained which form Equation (20). With *s* being the stress, *V* is the volume of the gel and $V_{0}$ is the volume of the referential state.

(20) $$A\nu \left(\lambda_{1}^{2}-1\right)+\left(\frac{V}{V_{0}}\right)\log\left[1-{\left(\frac{V}{V_{0}}\right)}^{-1}\right]+1+\left(\chi_{0}-\chi_{1}\right){\left(\frac{V}{V_{0}}\right)}^{-1}+2\chi_{1}{\left(\frac{V}{V_{0}}\right)}^{-2}=0,\frac{1}{\lambda_{3}}\left[N\nu \left(\lambda_{3}^{2}-1\right)+\left(\frac{V}{V_{0}}\right)\log\left[1-{\left(\frac{V}{V_{0}}\right)}^{-1}\right]\right]+\frac{1}{\lambda_{3}}\left[1+\left(\chi_{0}-\chi_{1}\right){\left(\frac{V}{V_{0}}\right)}^{-1}+2\chi_{1}{\left(\frac{V}{V_{0}}\right)}^{-2}\right]=\frac{s\nu }{k_{B}T}$$

### 4.4 Constitutive models for MRFs

In MRFs, rheology is crucial due to their two-phase nature. Carbon Iron Powder (CIP) is widely used for the initial phase of MRFs, due to its high saturation magnetization and wide availability [199]. The rheology affects the way the fluids are applied in practical settings. Experimental studies predominantly focus on shear stress and stress rate as key descriptors of the rheological properties of MRFs [200]. Various equations can be found for the shear stresses, all including different magnetic properties. This section will summarize the most prominent constitutive equations for MRFs based on [128].

The first is a traditional finite element model, which includes nonlinearity and saturation of the magnetization of particles integrated. In this model, MRFs have been simulated as chains of infinite length. The chains consist of aspherical magnetizable particles whose alignment depends on the applied direction of the magnetic field. The equation is given as Equation (21) with *γ* being the shear strain, *F* being the chain tension, and *W* being the radius of the particles. For the entire derivation of this equation refer to [128].

(21) $$\tau =\frac{\gamma }{1+\gamma^{2}}\frac{F}{\pi W^{2}}$$

A micro-macro model has been developed, based on a single-chain model. This model makes use of the mean magnetization approximation and can be found in Equation (22). In addition to this, a normal distribution is followed for the inclining angles of the evolving particle chains. With $\tau_{0}$ being the shear stress of the MRF without magnetic field applied, *A* is a constant, $\mu_{0}$ is the Permeability of vacuum, *ϕ* is the particle volume fraction, *R* is the core radius, *χ* is the magnetic susceptibility of magnetic particle, *H* illustrates the intensity of the magnetic field, $t_{h}$ demonstrates the thickness of the non-ferro-magnetic coat, and *δ* is the distance between two particles. For the second part of the equation: *θ* is the incline angle of the chain, *μ* is the mean, and *σ* is the standard deviation. For the entire derivation of Equation (22) refer to [201].

(22) $$\tau =\tau_{0}+\frac{A\mu_{0}\phi R^{3}\chi^{2}H^{2}}{{\left(2R+2t_{h}+\delta \right)}^{3}}\cdot \int_{-\frac{\pi }{2}}^{\frac{\pi }{2}}\frac{\left(5\cos^{2}\theta -1\right)\cos^{4}\theta \sin\theta }{\sqrt{2\pi }\sigma }e^{-\frac{{\left(\theta -\mu \right)}^{2}}{2\sigma^{2}}}\;d\theta$$

To adjust Equation (22) for a more exact dipolar model, but without using the approximation that the size of the particles is much smaller than the distance between the particles, yields Equation (23). In this equation, *n* is the number of particles and $r_{j}$ is the particle radius. Again the distribution of the inclining angles of the particle chains follows a normal distribution [202].

(23) $$\tau =\tau_{0}+\sum_{j=1}^{n-1}\frac{\mu_{0}\phi H^{2}\chi^{2}R^{3}(2R+\delta )}{r_{j}^{4}}\cdot \int_{\frac{\pi }{2}}^{\frac{\pi }{2}}\left[\left(1-5\cos^{2}\theta \right)-\frac{\chi R^{3}\left(1+4\cos^{2}\theta \right)}{3r_{j}^{3}}\right]\times \frac{\sin\theta }{\sqrt{2\pi }\sigma }e^{-\frac{{\left(\theta -\mu \right)}^{2}}{2\sigma^{2}}}\;d\theta$$

Equation (24) can be used to predict the yield shear stress of MRFs if fitting rate parameters are included. In Equation (24) *λ* is the rate parameter, *φ* is the volume fraction of magnetic particles in the MRFs, *r* is the radius of the magnetic particle, and *B* is the magnetic flux density [203].

(24) $$\tau =\frac{\varphi \pi^{2}r\chi^{2}B^{2}}{72\mu_{0}\delta }\lambda \left\{\frac{1}{3}+\frac{3\lambda \times e^{-\lambda \pi /2}-\lambda^{2}}{12\left(\lambda^{2}+9\right)}-\frac{\lambda \times e^{-\lambda \pi /2}+\lambda^{2}}{4\left(\lambda^{2}+1\right)}\right\}$$

An initial chain model was also established by assuming a normal distribution for the inclining chain angles, which results in Equation (25). In this model, *ξ* has been an additional term as a function of *θ* [204].

(25) $$\tau =\frac{\varphi AR^{3}\chi^{2}B^{2}}{\mu_{0}{\left(1+\chi_{1}\right)}^{2}(2R+\delta +2t)}\xi$$

A compact two-column model is given in Equation (26), where *ψ* is an additional term as a function of *θ*. In this model, the exponential distribution was applied to describe the distribution of the inclining chain angles, for the complete derivation see [205].

(26) $$\tau =\frac{\phi r^{3}\chi^{2}B^{2}}{3\mu_{0}{\left(1+\chi \right)}^{2}{\left(2r+2t+\delta \right)}^{3}}\psi$$

A profound theoretical analysis of the microstructure of MRFs found the following Equation (27), where *a* is the particle radius, *k* is a parameter to describe the effect of inhomogeneity of CIP particles, and *σ* is the deviation angle of a particle. This model is based on the structure evolving in a stable hexagonal close-packed structure from a microscopic perspective. Equation (27) is also the only model which assumes multiple chains of particles [206].

(27) $$\tau =\frac{4H^{2}a^{3}\varphi \chi^{2}\mu_{0}}{3k{\left(2a+2t+\delta \right)}^{3}}f(\sigma )$$

ERFs are similar to MRFs, Kumar [207] provides an interesting continuum-based method for the modelling of electro-magneto-rheological fluids (EMRFs) which are exposed to an electromagnetic field. In this approach the principles of physics and thermodynamics are combined, resulting in a generalized constitutive model. The continuum-based model attempts to generalize the deformations of a fluid continuum to EMR. The generalization is something that is quite in contrast with existing works performed on ERFs and MRFs.

Furthermore, Table 3 gives an overview of all the types of constitutive equations being discussed in this chapter regarding the four groups of Smart materials: SMAs, SMPs, Hydrogels and MRFs. It is important to note that the equations that are included in this chapter are chosen due to their fundamental role in the creation of specific models for different applications or since they are most commonly used as models. Nevertheless, there are many more models which could be used, all depending on the application at hand or the information or data needed to gather.

**Table 3:** Summary constitutive models.

Type	Equation	Model
SMA	Eq. (3)	Adjusted Brinson Model [172]
	Eq. (5)	Boyd and Lagoudas - phenomenological model [173]
	Eq. (7)	Auricchio and Petrini - phenomenological model [176]
	Eq. (9)	ZM phenomenological model [177]

SMP	Eq. (10)	Basic viscoelastic model [183]
	Eq. (11)	Thermoviscoelastic models [184]
	Eq. (12)	Fractional viscoelastic constitutive model [185]
	Eq. (13)	Classic phenomenological constitutive model [187]
	Eq. (14)	Phenomenological models of SMP considering rate effect [188]

Hydrogel	Eq. (16)	Neutral Gel model [192]
	Eq. (17)	Salt concentration gel model [194]
	Eq. (18)	pH-sensitive gel model [196]
	Eq. (20)	Temperature sensitive Gel model [198]

MRF	Eq. (21)	Finite-element microscopic model [128]
	Eq. (22)	Micro-Macro model [201]
	Eq. (23)	Magnetic-dipoles-based micro-macro model [202]
	Eq. (24)	Yield shear stress model [203]
	Eq. (25)	Initial tilt chain model [204]
	Eq. (26)	Compact two-column model [205]
	Eq. (27)	Micro model based on hexagonal closepacked structure [206]

## 5 Discussion and conclusion

Various modelling techniques, notably FEA, multiscale modelling, and PFM are used in smart materials analysis. PFM is significant in SMAs due to phase transitions altering microstructural boundaries, affecting FEA efficacy. Constitutive models are pivotal, especially in SMAs, forming the basis for modern modelling approaches tailored to specific applications. SMAs undergo phase transformations in response to changes in temperature or stress. Constitutive models need to account for the thermomechanical coupling inherent in these transformations. The relationship between temperature and mechanical deformation is crucial for accurately describing SMA behaviour. The constitutive models discussed for SMPs can be widely applied, however, it is worth mentioning that the various working conditions do affect the mechanical characteristics of SMPs differently. The models provided here, also for hydrogel, are most of the time limited to 1D or 2D cases. This makes the implementation of FEM or other numerical methods very important. Since some of these models are preliminary and need to be further developed. In addition to this, these models are discussed under the assumption that the intrinsic mechanisms of thermal-induced SMPs are similar to most SMPs. The hydrogel models developed can accurately depict and predict the deformation of the different types of hydrogel with complex shapes. It must be noted that all of the equations given are based on the Neo-Hookean model. However, other hyperelastic materials models could be used to obtain the constitutive equations for hydrogel above, such as the Ogden Model or the Mooney-Rivlin model. Each of these models is more accurate for a different strain level, for example, Mooney–Rivlin accurately describes the stresses for small strains [208]. Furthermore, the models are based on the neutral gel model, which combines coupling and diffusion. The other hydrogel models are based on adding specific terms which are required for the free energy function. Lastly, as mentioned before, the concentration *C* is dependent or independent of J. It is problematic to implement the independent relation in ABAQUS, this could be solved later with future studies. It can be seen that most of the equations for MRFs are derived from one another and the biggest difference is in the distribution used for the inclining chain angles. In the literature, no consensus has been reached over the distribution to be used. The above-mentioned microscopic models are based on the evolution of the microstructure of MRFs. These models are independent of experimental data, which is contrary to macroscopic models. The majority of the rheology of MRFs is determined by the microscopic behaviour, which is the reason why these equations are included in this analysis. In particular, models based on micromechanics include influencing factors such as particle size and magnetic permeability, making them more complex than the macroscopic model. Nevertheless, the relationship between macroscopic and microscopic models has not been thoroughly investigated in the literature.

## 6 The future of model-based design of mechanically intelligent structures

**Figure 5:**
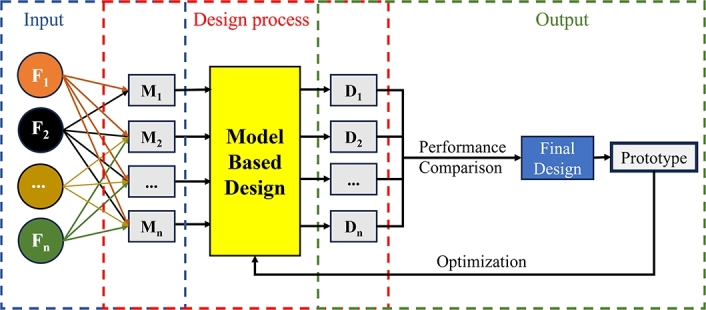
Flowchart of the purpose of the model-based design with functions as input and a prototype as output.

The optimization of model-based design has emerged as a critical approach for unlocking the full potential of smart structures. By utilizing numerical models, researchers and engineers can simulate and predict the behaviour of smart materials under different conditions, enabling them to optimize their design parameters. The process of model-based design involves employing simulation tools and numerical methods to explore a vast design space, ultimately identifying the most efficient and effective solutions. Constitutive models are needed to accurately depict the nonlinear behaviour of a smart material. Each type of smart material has its respective type of constitutive equation based on a specific free energy function. Each constitutive equation includes the parameters found by experimental data, resulting in less accuracy to some extent. Each constitutive equation depends on the application and the required phenomena should be studied. The mechanically intelligent structure is an adaptive structure actuated by multiple stimuli. A desire for a functional mechanically intelligent structure requires a complex model for design optimization. To improve smart materials-based design, proper selection of suitable simulation tools and constitutive equations are required to eventually reach the model-based design framework. The flowchart of model-based design framework is shown in Fig. 5. A set of functions ($F_{i}$) as input, such as scale, stiffness or strength together with a variety of smart materials ($M_{i}$) lead with model-based design to a set of designs ($D_{i}$) of which a final design is chosen due to performance comparison and then leading to a working prototype.

For future work, coupling effects between different physical phenomena need to be discovered. Modelling the interactions between different phenomena has been a challenge with a majority of constitutive models. Smart materials often link the coupling of different phenomena, the modelling of these materials requires a thorough grasp of the underlying mathematical and physical formulations. Design optimization can then be reached since multiple options can be considered, while not being limited to a specific material. Additionally, more general models that capture stimuli-responsive behaviour could be derived for design purposes. Furthermore, the modelling of smart materials is based on static analysis in this study, thereby neglecting dynamic characteristics. However, work on the dynamic behaviour is currently a hot topic and deserves a new literature review. Consequently, future modelling approaches will incorporate the transient behaviours of smart materials to enhance accuracy and applicability.

## CRediT authorship contribution statement

**Qianyi Chen:** Writing – review & editing, Writing – original draft. **Tarish Kalpoe:** Formal analysis. **Jovana Jovanova:** Supervision.

## Declaration of Competing Interest

The authors declare that they have no known competing financial interests or personal relationships that could have appeared to influence the work reported in this paper.
